# RNA-SSNV: A Reliable Somatic Single Nucleotide Variant Identification Framework for Bulk RNA-Seq Data

**DOI:** 10.3389/fgene.2022.865313

**Published:** 2022-06-30

**Authors:** Qihan Long, Yangyang Yuan, Miaoxin Li

**Affiliations:** ^1^ Zhongshan School of Medicine, Sun Yat-Sen University, Guangzhou, China; ^2^ Center for Precision Medicine, Sun Yat-Sen University, Guangzhou, China; ^3^ Center for Disease Genome Research, Sun Yat-Sen University, Guangzhou, China; ^4^ Guangdong Provincial Key Laboratory of Biomedical Imaging and Guangdong Provincial Engineering Research Center of Molecular Imaging, The Fifth Affiliated Hospital, Sun Yat-sen University, Zhuhai, China; ^5^ Key Laboratory of Tropical Disease Control (SYSU), Ministry of Education, Guangzhou, China

**Keywords:** cancer, somatic mutation, RNA, RNA-Seq, machine learning, RNA-SSNV

## Abstract

The usage of expressed somatic mutations may have a unique advantage in identifying active cancer driver mutations. However, accurately calling mutations from RNA-seq data is difficult due to confounding factors such as RNA-editing, reverse transcription, and gap alignment. In the present study, we proposed a framework (named RNA-SSNV, https://github.com/pmglab/RNA-SSNV) to call somatic single nucleotide variants (SSNV) from tumor bulk RNA-seq data. Based on a comprehensive multi-filtering strategy and a machine-learning classification model trained with comprehensively curated features, RNA-SSNV achieved the best precision–recall rate (0.880–0.884) in a testing dataset and robustly retained 0.94 AUC for the precision–recall curve in three validation adult-based TCGA (The Cancer Genome Atlas) datasets. We further showed that the somatic mutations called by RNA-SSNV tended to have a higher functional impact and therapeutic power in known driver genes. Furthermore, VAF (variant allele fraction) analysis revealed that subclonal harboring expressed mutations had evolutional selection advantage and RNA had higher detection power to rescue DNA-omitted mutations. In sum, RNA-SSNV will be a useful approach to accurately call expressed somatic mutations for a more insightful analysis of cancer drive genes and carcinogenic mechanisms.

## Introduction

Cancer is the leading cause of death and an important barrier to increasing life expectancy (Sung et al., 2021). According to GLOBOCAN 2020 estimates of cancer incidence and mortality, 19.3 million new cancer cases and 10.0 million cancer deaths occurred in 2020 (Sung et al., 2021). Somatic mutations are usually induced by environmental factors, and it is well known that their accumulation with aging and evolution in human cells will lead to malignant transformation and eventually cancer (Watson et al., 2013). Thus, comprehensive somatic mutation identification in cancer such as the Catalogue Of Somatic Mutations In Cancer (Tate et al., 2019) (COSMIC database) can help characterize its genomic complexities (Watson et al., 2013) and discover oncogenic mutations and driver genes which significantly influence cancer development (Bailey et al., 2018). Furthermore, person-level somatic mutations also have their own oncogenic and therapeutic implications in multiple cancers (lung cancer (Skoulidis and Heymach,2019), bladder cancer (Cazier et al., 2014; Wen et al., 2021), and glioblastoma (Lin et al., 2021; McDonald et al., 2015)), targeting the corresponding mutant proteins or pathways. Currently, most somatic mutation identification studies were based on DNA-level, actionable practices in somatic mutation detection within whole-genome or whole-exome sequencing data have been developed to facilitate precision oncology (Xiao et al., 2021).

Mutations within exons are supposed to be transcribed into RNA, and be reflected in the translated protein. However, many DNA mutations within exons were not found in RNA because they were located in the non-transcribed allele or had no or low expression (O Brien et al., 2015). Yizhak et al. (2019) reported that 65% of DNA somatic mutations within 243 TCGA tumor samples were not detected in RNA. Rashid et al.(2014) found that only 27% of mutated alleles got expressed in multiple myeloma. The significant lack of DNA mutations in RNA indicated that not all DNA mutations have certain effects finally. RNA can be a reliable source to distinguish mutations that have been expressed to affect cellular functions. Although RNA-seq is mainly used for gene expression and fusion discoveries in clinical oncology (Wang et al., 2020), previous studies showed that calling genomic variants in expressed exons using RNA-seq data was feasible and cost-effective (Chepelev et al., 2009; Cirulli et al., 2010; Gonorazky et al., 2019; Piskol et al., 2013; Quinn et al., 2013). The advantages included making the most abundant RNA-seq data resources and discovering rare somatic mutations with the low-level DNA allele fraction at higher sequencing depths in sufficiently expressed genes (Chepelev et al., 2009; Cirulli et al., 2010; Gonorazky et al., 2019; Piskol et al., 2013; Quinn et al., 2013; Liu et al., 2014). However, calling somatic mutations within RNA-seq data was challenging compared with calling variants in WES data. The main challenge was the high false-positive rate, deriving from errors during reverse transcription, misalignment near splicing junctions (exon ends), RNA editing, and modification during post-transcriptional processing (Cirulli et al., 2010; Xu, 2018). Multiple RNA somatic mutation calling tools and pipelines have been developed to remove these false-positive calls, which can be divided into two categories: statistical filtering strategy-based (García-Nieto et al., 2019; Neums et al., 2018; Yizhak et al., 2019) and machine learning–based approaches (Muyas et al., 2020; Sheng et al., 2016). For instance, GLMVC (Sheng et al., 2016) calls RNA somatic mutations based on a bias-reduced generalized linear model trained by the characteristics of RNA-seq data. VaDiR (Neums et al., 2018) integrates results from three variant callers and produced higher precision results through consensus combination but sacrificed sensitivity. RNA-MuTect (Yizhak et al., 2019) comprehensively filtered mutations within artifact sites and achieved optimal performance. RF-RNAMut (Muyas et al., 2020) utilized a machine learning model to distinguish somatic variants from germline variants identified in RNA-seq data. Although existed tools have their advantages and highlights, they had their limitations: (1) unsatisfying precision–recall performance with the maximum reported precision–recall to be 0.87–0.72 (Yizhak et al., 2019), (2) required restricted resources such as DNA and RNA panel of normal (PoN) calls from ∼6500 GTEx samples to achieve a desired result (Yizhak et al., 2019), and (3) model not specifically trained to recognize excessive artifacts in RNA but to identify germline mutations as negative (Muyas et al., 2020).

Here, we introduce a framework named RNA-SSNV (https://github.com/pmglab/RNA-SSNV). It is a unified framework containing a universal pipeline to call RNA somatic single nucleotide variants from the combination of tumor RNA-seq and normal WES data, a multi-filtering strategy to remove doubtful calls with little loss of sensitivity and a supervised machine learning model to identify somatic mutations and artifacts. Our framework achieved the best overall performance for precision and recall, requiring only public reference resources. To validate the generalization performance of our framework, we utilized RNA-SSNV within TCGA lung squamous cell carcinoma (LUSC), bladder urothelial carcinoma (BLCA), and glioblastoma multiforme (GBM) independent datasets. RNA-SSNV achieved similar performance in the area under curve (AUC) for the precision–recall curve with 0.94 for all three datasets. Given its high precision–recall performance, RNA-SSNV will help exploit expressed somatic variants, further extend the range of RNA-seq applications and make full use of abundant RNA-seq data resources.

## Materials and Methods

### Framework Overview

**FIGURE 1:**
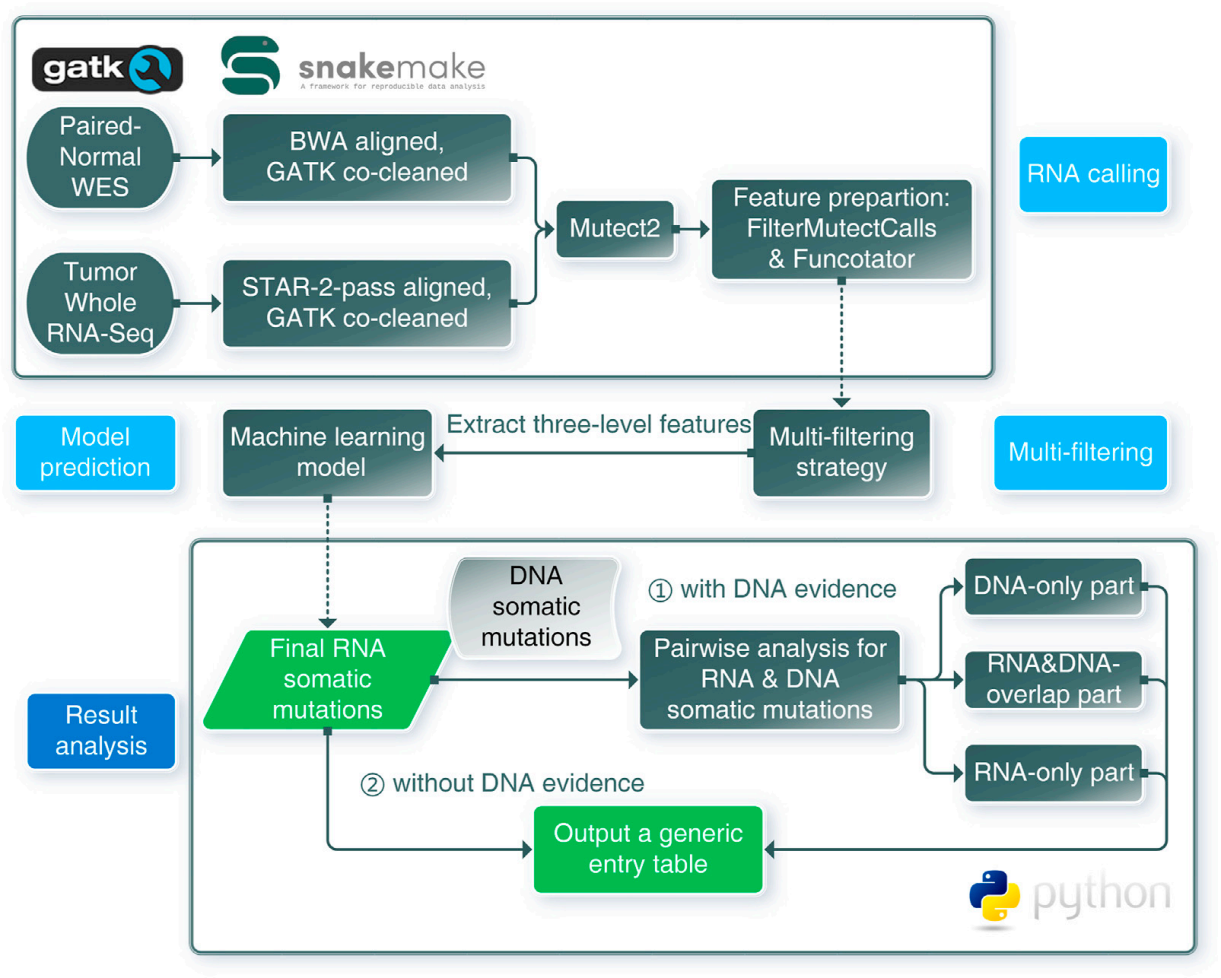
Schematic overview of the framework for RNA somatic mutation identification. RNA calling: RNA-seq and WES data were aligned and co-cleaned accordingly. Mutect2 was used to conduct RNA somatic calling with paired tumor RNA-seq and normal WES data. Features were extracted from outputs of FilterMutectCalls and Funcotator. Multi-filtering: multi-filtering strategy was conducted in Mutect2 called mutations by removing multiallelic, RNA-editing, immunoglobin, and HLA sites. Model prediction: using the trained model, mutations with extracted features were predicted as positive or negative, only positives were regarded as reliable mutations. Result analysis: pairwise analysis can be conducted when DNA evidence was available. RNA-SSNV will output a generic entry table containing all features and predicting information to facilitate downstream analysis.

Our RNA somatic single nucleotide variant identification framework (RNA-SSNV) consists of three major components, including a RNA somatic mutation calling step, a multi-step filtering process and a machine-learning based prediction (Figure 1). The underlying hypothesis of RNA-SSNV is that RNA-specific mutations have unique biological and technique features; thus, a comprehensive filtration process and a machine learning model based on these features can substantially improve the accuracy of RNA somatic mutation calling.

### Datasets

Our datasets were retrieved from GDC, which had harmonized pipelines (https://docs.gdc.cancer.gov/Data/Introduction/) to generate RNA-seq and DNA-seq data. All RNA-seq datasets were aligned to GRCh38 build using a two-pass method with STAR, which required preprocessing before mutation calling. All DNA-seq datasets were aligned to the GRCh38 reference using bwa (Li and Durbin, 2009) and co-cleaned using the GATK toolkit (McKenna et al., 2010), which can directly be utilized in mutation calling.

We chose the TCGA lung adenocarcinoma (LUAD) cohort as the training dataset that contained the largest patient scale (511) compared with other available cancer cohorts. Our training dataset comprised paired tumor RNA-seq and tumor/normal WES data derived from 511 LUAD patients, which simultaneously generated DNA and RNA somatic mutations. Our independent validation datasets comprised paired tumor RNA-seq and normal WES data derived from 498 LUSC, 441 BLCA, and 198 GBM patients, for which we called RNA somatic mutations to get validating records.

### Mutation Calling

Theoretically, calling somatic mutations within RNA-seq data can be easily conducted using callers designed for DNA. Haplotype-based callers (GATK Mutect2 (Benjamin et al., 2019; Cibulskis et al., 2013), TNscope) had been proven to outperform position-based variant callers due to their inherent technical advantage in complex variants and high mutation loading regions (Pei et al., 2020; Xu, 2018). In addition, we queried the TCGA helpdesk and learned that our RNA-seq data (TCGA LUAD, LUSC, GBM, and BLCA projects) were sequenced by the UNC center using poly-T mRNA enriching strategy, which indicated that only transcribed exon regions (GENCODE v22 annotated exon regions) within mature mRNA can be sequenced (Kukurba and Montgomery, 2015) and our paired normal targeted capture exome sequencing (WES) data had a canonical target region (Agilent SureSelect TargetInterval). Thus, we chose to utilize Mutect2 to perform somatic variant calling and only retain mutations within targeted coding regions (overlap of exons and WES targets).

Normally, our STAR-2-pass aligned RNA-seq data required a co-cleaning process to conduct mutation calling. Following GATK recommended procedures (RNAseq Best Practice), our aligned RNA-seq bam was passed to the MarkDuplicates tool to identify duplicate reads and help remove PCR-related artifacts. Next, SplitNCigarReads hard-clipped and reformat some alignments which span introns causing large-scale mistaken indels. Finally, it shall undergo base quality recalibration conducted by BaseRecalibrator and ApplyBQSR to detect and correct patterns of systematic errors in the base quality scores.

After obtaining analysis-ready bam files, we utilized Mutect2 to call RNA somatic mutations from paired tumor RNA-seq and normal WES data, DNA somatic mutations from tumor and normal WES data. For the TCGA LUAD training set, we called RNA and DNA somatic mutations to help construct the training dataset. For TCGA LUSC, GBM, and BLCA validation sets, calling RNA somatic mutations were sufficient to validate our framework’s performance. For DNA somatic mutations omitted in RNA which required verification, we applied the force-calling mode in Mutect2 to retrieve their RNA mutational status. Finally, we utilized FilterMutectCalls to generate quality information as training features and assess the performance for Mutect2’s default filtering, Funcotator to annotate variants and facilitate downstream analysis.

### Multi-Filtering Strategy

Before model training or predicting, RNA somatic mutations shall be comprehensively filtered to remove known possible artifacts (García-Nieto et al., 2019; Yizhak et al., 2019). Our multi-filtering strategy included removing multi-allelic mutations, RNA-editing sites, IgG, and HLA regions. For multi-allelic mutations, we removed mutations containing three or more allele types to avoid misaligning artifacts. For RNA editing events, we combined A-to-I RNA editing information from the REDIportal (Mansi et al., 2021) database and further editing information from the DARNED (Kiran et al., 2013) database. We removed all mutations which located in the union set of RNA editing events to prevent these false-positive calls. For IgG regions, we removed mutations falling into IgG genes to avoid noisy alignments (Ye et al., 2013). For HLA regions, we removed the HLA mutations in chromosome 6 which contained a high density of germline variants (Buhler and Sanchez-Mazas, 2011).

### Construct a Training Dataset

For all TCGA projects involved in our study, the GDC Data Portal (https://portal.gdc.cancer.gov/) already provided open-access DNA somatic mutations detected by four different callers MuSE, MuTect2, SomaticSniper, and VarScan (Ellrott et al., 2018) with stringent thresholds. Using the GDC MAF Concatenation Tool (https://github.com/wwysoc2/gdc-maf-tool), we combined the curated mutations from four callers, and constructed a union set of all available DNA somatic mutations for each cancer type (LUAD, LUSC, BLCA, and GBM) to maximize the sensitivity. In addition, given that GDC somatic variant calling pipeline had strict criteria leading to the loss of some true positive somatic mutations, we called our own DNA somatic mutations using raw sequencing data and retrieved GDC-omitted DNA somatic mutations.

**FIGURE 2:**
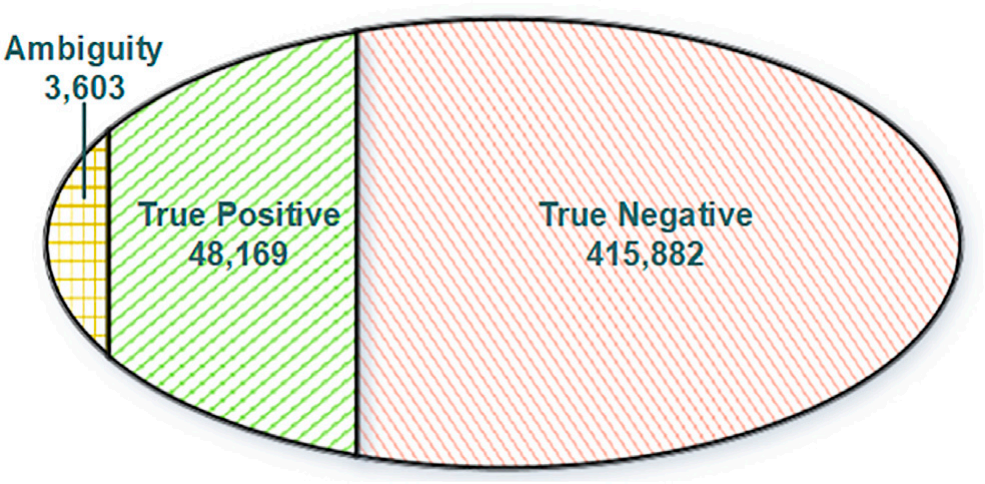
Venn diagram of training dataset categories. True positive: RNA somatic mutations overlapping with GDC mutations. Ambiguity: RNA somatic mutations overlapping with GDC omitted somatic mutations. True negative: RNA somatic mutations without DNA support.

Normally, variations in DNA will be passed and presented in RNA through transcription. Reciprocally, any RNA somatic mutations presented in DNA should be true positive since they have got evidence from DNA. Moreover, other RNA somatic mutations lacking support from DNA will be regarded as true negative. To construct a reliable training dataset for model training, we split our RNA somatic mutations into three categories (Figure 2) based on evidence from the GDC database and GDC-omitted DNA somatic mutations. Finally, based on the information from FilterMutectCalls output and annotation information of Funcotator, we systematically extracted features for each training record with three categories: variant, genotype, and annotation levels (Supplementary Table S1).

### Performance Metrics

Due to the extreme distribution bias for true positive and true negative classes (TP : TN = 1:8), our main purpose was to identify true positive RNA somatic mutations correctly. We chose precision, recall, F1 scores, and areas under the precision–recall curve (PR-AUC) as major performance metrics in our study because they are insensitive to class imbalance. Other metrics derived from the confusion matrix (Table 1) were also introduced for evaluation.

$$\text{Precision}=\frac{\text{True Positive}}{\text{True Positive}+\text{False Positive}},$$

$$\text{Recall}=\frac{\text{True Positive}}{\text{True Positive}+\text{False Negative}},$$

$$\text{F}1=2\text{∗}\frac{\text{Precision ∗ Recall}}{\text{Precision}+\text{Recall}},$$

$$\text{False positive rate}=\frac{\text{False Positive}}{\text{False Positive}+\text{True Negative}},$$

$$\text{False negative rate}=\frac{\text{False Negative}}{\text{False Negative}+\text{True Negative}},$$

$$\text{True negative rate}=\frac{\text{True Negative}}{\text{False Negative}+\text{True Negative}}.$$

**TABLE 1:** Confusion matrix demonstration.

	Predicted condition		
	Label	Positive	Negative
True condition	Positive	True positive	False negative
	Negative	False positive	True negative

### Model Training and Validation

Records within the training dataset were split into training and testing subsets (9:1). We utilized the training subset for model parameter tuning, feature selection, and model training. For the testing subset, we utilized them for testing the model’s generalization performance.

To handle the imbalanced distribution for TP and TN classes, we chose a weighted random forest classifier (RandomForestClassifier, scikit-learn 0.24.2) to reduce the bias by assigning inversely proportioned weights to different classes (Zhu and Pierskalla, 2016). First, we utilized recursive feature elimination with 10-fold cross-validation (RFECV, scikit-learn 0.24.2) to select optimal features. Second, we utilized a 10-fold cross-validated grid-search over a parameter grid (GridSearchCV, scikit-learn 0.24.2) to fine-tune optimal parameters (max_depth, min_samples_split, min_samples_leaf, max_features, etc.). Finally, we constructed a machine learning model for RNA somatic mutation identification with optimal features and parameters, and applied it in testing subset to assess its generalization performance.

Following the procedures mentioned earlier, we conducted somatic single nucleotide variants calling in LUSC, BLCA, and GBM cohorts, utilized a multi-filtering strategy and built validation datasets based on extracted features. We applied our discriminant model in these validation datasets and retrieved assessing metrics to further demonstrate the generalization performance.

Also, we validated the necessity of introducing a new training dataset from another cancer type. We added the GBM dataset into the initial training dataset and constructed a new random-forest classifier. After retrieving and assessing metrics for the new random-forest classifier within LUSC and BLCA independent validation datasets, we compared them with our initial model’s performance.

### Model Interpretation and Visualization

We utilized impurity-based feature importance for tree-based machine learning models to help interpret features’ contributions within our model. The higher its contribution, the more important the feature. Impurity-based feature importance (Gini importance) is computed as the total reduction of the criterion brought by that feature and retrieved through our model’s attribute feature_importances_. Because traditional feature importance mainly focused on overall model interpretation, we also introduced the SHAP (SHapley Additive exPlanations, https://github.com/slundberg/shap) (Lundberg and Lee, 2017) python package to help visualize prediction (Lundberg et al., 2018) and provide local explanations (Lundberg et al., 2020). We provided feature contributions calculated by SHAP for predicted probability and conducted a single prediction’s visualization by invoking the force_plot function. We also investigated the feature contributions of the training dataset. We calculated and visualized the sum of SHAP value magnitudes by summary_plot function in SHAP to show the distribution of each feature’s impacts on the model output (lift or lower prediction probability).

### Whole Framework Implementation

We built our whole framework using Snakemake (Köster and Rahmann, 2012) and class-oriented python scripts. Snakemake (https://github.com/snakemake/snakemake) was applied to manage standard bioinformatic workflows involved in this study (co-cleaning, calling, and annotation) and conduct task auto-management without complicating shell scripts. Function-oriented python scripts contained feature extraction, model training and testing, and model utilizing function. Both Snakemake-based workflows and python scripts were available within our project repository (https://github.com/pmglab/RNA-SSNV), which helped create reproducible analysis.

### Analyze RNA Mutations With DNA Evidence

**FIGURE 3:**
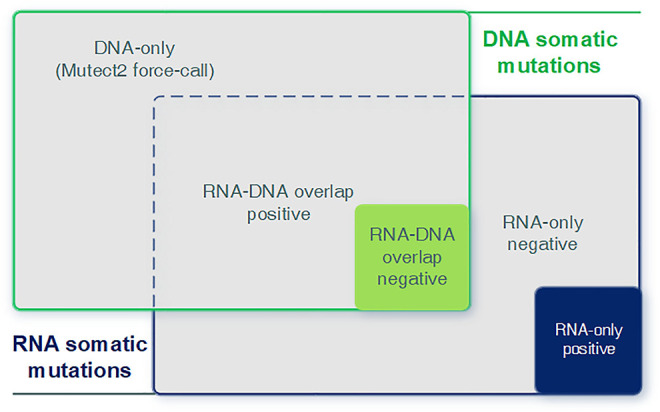
Graphical introduction for the DNA-only, DNA–RNA overlap, and RNA-only parts. Graphical introduction for detailed combination of RNA and DNA somatic mutations. DNA-only: DNA somatic mutations not detected (expressed) in RNA. RNA–DNA overlap: somatic mutations detected in both RNA and DNA. RNA-only: RNA somatic mutations without any DNA evidence.

We integrated predicted RNA somatic mutations with known DNA mutations to analyze the relevance between RNA and DNA. We examined their intersectionality and split them into three parts (RNA–DNA overlap, DNA-only, and RNA-only) and two sub-categories (positive and negative class, Figure 3). Each part and sub-category had its biological implication and interpretation requiring further investigation. The RNA–DNA overlap part stood for RNA mutations with DNA evidence support. DNA-only part stood for DNA mutations not detected in RNA, and we utilized the Mutect2 force-call mode to inspect their coverage status in RNA. RNA-only part stood for RNA mutations not detected in DNA, and most of them were artifacts due to lack of DNA evidence or low sequence qualities.

Cancer driver genes were under positive selection during tumorigenesis (Martinez-Jimenez et al., 2020). Here, we focused on cancer-specific driver genes (https://www.intogen.org/) to explore their enrichment patterns (number distribution, functional impact, and therapeutic power) between expressed (RNA–DNA overlap part) and un-expressed (DNA-only part) somatic mutation panels. For pathogenicity prediction, Combined Annotation–Dependent Depletion (CADD) (Rentzsch et al., 2019), Eigen Principal Components (Eigen-PC) (Ionita-Laza et al., 2016), Polymorphism Phenotyping version 2 (PolyPhen-2) (Adzhubei et al., 2010), Protein Variation Effect Analyzer (PROVEAN) (Choi et al., 2012), UMD-Predictor (Ioannidis et al., 2016), Rare Exome Variant Ensemble Learner (REVEL) (Frederic et al., 2009), and Sorting Intolerant From Tolerant (SIFT) (Ng, 2003) were top-performing prediction tools on somatic variants (Suybeng et al., 2020). Thus, we used the dbNSFP v4.1a (Liu et al., 2020) database to annotate missense mutations with the aforementioned prediction scores. The chi-squared test was used to calculate the significance (*p*-value) of enriched distribution and odds ratio (OR). A two-sided independent *t*-test was used to determine the significance (*p*-value) of the difference between the means of two prediction groups.

We also conducted an analysis of transcriptome-wide allele-specific expression (ASE) to identify ASE events in somatic mutations and their impacts on gene expression which affected carcinogenesis. We chose cases containing both tumor and paired-normal RNA-seq data from LUSC and BLCA cohorts (LUSC: 49 cases, BLCA: 19 cases), and curated their gene expression profiles from the UCSC Xena database (https://xena.ucsc.edu/). Then, we chose only heterozygous SNVs in both tumor RNA-seq and WES data (RNA–DNA overlap part), and implemented chi-squared tests on the RNA and DNA allelic depths with a significance cutoff of *p*-value 0.01 to identify somatic SNV ASEs (Heap et al., 2010; Liu et al., 2016). Finally, we compared the TPM value of tumor and paired-normal samples of cases harboring the somatic SNV ASEs to examine the alteration of total gene expression, and defined the TPM fold change (FC) of 2 and 1/2 as the thresholds of upregulated and downregulated genes (Liu et al., 2018).

## Results

### General Performance of the Framework

**FIGURE 4:**
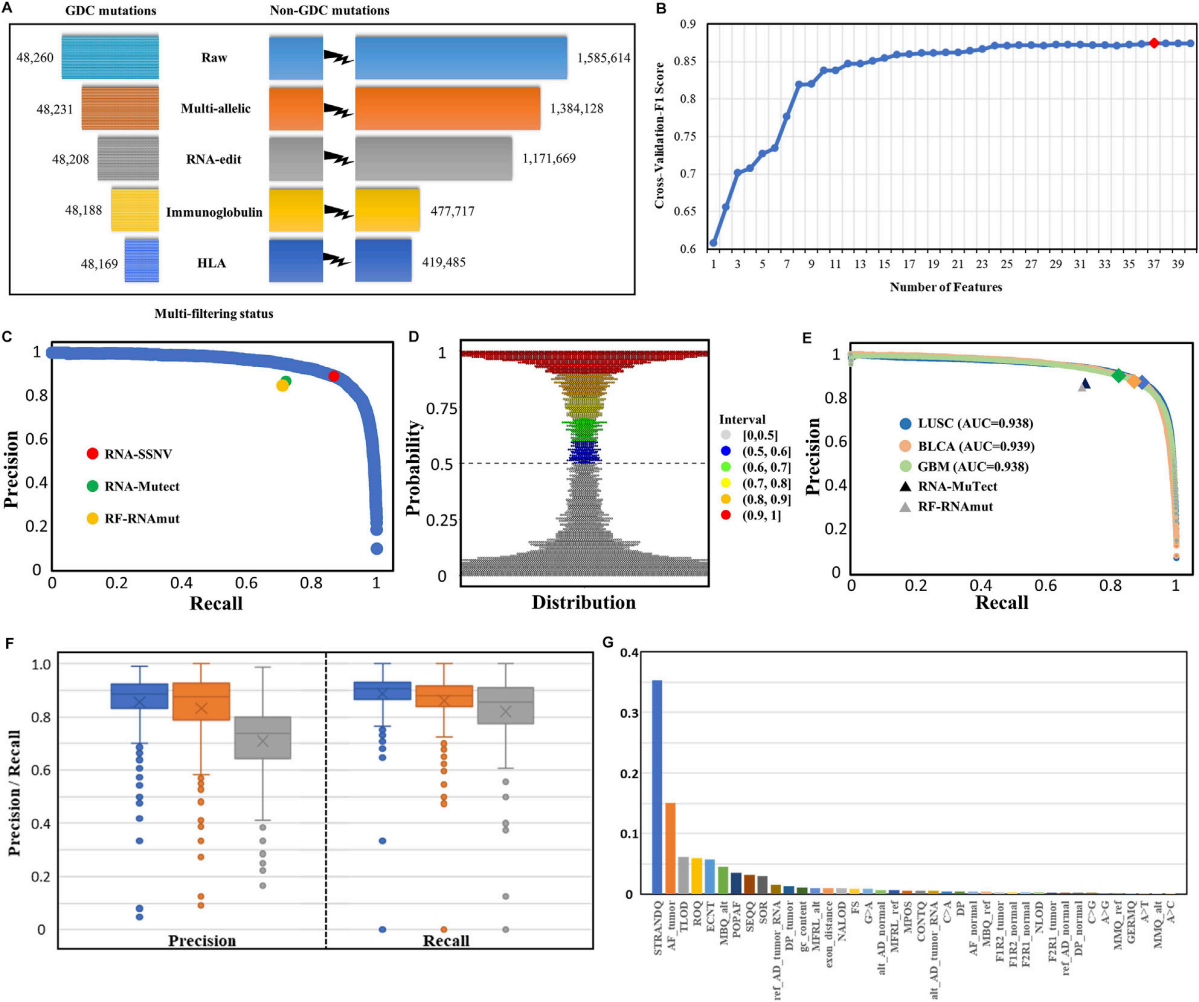
Multi-filtering strategy and machine-learning model performance in testing and validation datasets. **(A)** Loss of GDC mutations (true positive) and non-GDC mutations after the removal of multiallelic, RNA-editing, immunoglobulin, and HLA sites. **(B)** Change in cross-validated F1 score with the number of features decreasing using the Recursive Feature Elimination with Cross-Validation (RFECV) method. Initial number of features was 40 and each iteration removed one least important feature. **(C)** P–R (blue) curve for the testing dataset. RNA-SSNV achieved 0.880 precision and 0.884 recall rate (red point) in the testing dataset under the default 0.5 threshold. RNA-Mutect (green point) and RF-RNAmut (orange point) had reported precision–recall with 0.87–0.72 and 0.85–0.71, respectively. **(D)** Probability distribution of the predicted scores for the testing dataset. Most somatic mutation records were at the upper or lower ends of the plot, conforming a clear classification boundary. **(E)** P–R curves for independent validation datasets. P–R curves for LUSC (blue), BLCA (orange), and GBM (green) had identical 0.94 AUC. The peaks meant slightly different P–R performances for our model using the default 0.5 threshold in three datasets: LUSC (0.872–0.894), BLCA (0.876–0.870), and GBM (0.902–0.825). P–Rs for RNA-Mutect and RF-RNAmut were also used for comparison. **(F)** Precision and recall distribution for each case across three types of cancer (LUSC, BLCA, and GBM). Box plots showed median, 25th and 75th quantiles, outliers were presented as dots. **(G)** Relative importance distribution for each feature. Gini impurity-based feature importance values were normalized to sum to one.

After the initial RNA somatic mutation calling and multi-filtering step, we collected 467,654 mutations in the LUAD training dataset and 721,234, 323,323, and 126,449 mutations in LUSC, BLCA, and GBM independent validation datasets, respectively. To evaluate the effectiveness of multi-filtering strategy, we validated the loss of GDC mutations in the LUAD training dataset (Figure 4A) and LUSC, BLCA, and GBM independent validation datasets (Supplementary Figure S1). We found that the loss was negligible (0.1%), whereas the reduction of possible artifact calls was rather significant (70%); such preprocessing guaranteed a relatively pure mutation set for training and predicting. Furthermore, our framework’s built-in machine learning model was trained and fine-tuned by 10-fold cross-validation. In total, 37 features from three categories were kept for model training after feature selection conducted in the initial 40 features (Figure 4B). Finally, our framework achieved 88.0% precision and 88.4% recall rate within the testing dataset (Figure 4C), and other assessing metrics (Table 2) were also satisfying. For example, the false-positive rate was 0.014, the false-negative rate was 0.013, and the true-negative rate was 0.987. Moreover, most RNA somatic mutations were at the upper or lower ends of the bay plan plot according to the predicted probability distribution of the testing dataset (Figure 4D), which suggested a clear classification result.

**TABLE 2:** Confusion matrix for the holdout testing dataset.

	Predicted condition		
	Label	Positive	Negative
True condition	Positive	4,165	546
	Negative	566	41,129

To inspect the generalization performance of our framework, we applied our RNA somatic mutation discriminant model to three independent validation datasets. As a result, RNA-SSNV successfully discriminated GDC high confidence somatic variants from WES-targeted coding RNA mutations with significantly higher precision, recall, and PR-AUC (LUSC P–R: 0.872–0.894, BLCA P–R: 0.876–0.870, and GBM P–R: 0.902–0.825, Figure 4E), compared with other RNA somatic detection tools such as RNA-Mutect (Yizhak et al., 2019) (precision: 0.87, recall: 0.72) and RF-RNAmut (Muyas et al., 2020) (precision: 0.85, recall: 0.71). Specially, RNA somatic mutations within cancer-specific driver genes had better performance (LUSC P–R: 0.924–0.921, BLCA P–R: 0.929–0.896, and GBM P–R: 0.921–0.883) and they had higher coverages than total RNA somatic mutations (median sequencing coverages—LUSC overall: 42, driver: 60, two-sided independent *t*-test *p-*value: 1.06e-7; BLCA overall: 41, driver: 44, *p-*value: 7.35e-7; GBM overall: 46, driver: 76, *p-*value: 1.12 e-8). Thus, critical mutations within cancer driver genes can be reliably identified in RNA-seq data, which also guarantees our framework’s clinical value.

For case-level performance, as expected, LUSC and BLCA retained a median precision of 0.885 and 0.876 across cases, but GBM only reached 0.739 median precision (Figure 4F), contradicting its general precision of 0.902. Such contradiction was caused by four high-mutation-rate (harbored more than 100 DNA mutations) cases having high precision (>0.950). In contrast, most GBM cases had extremely low somatic mutation rates with less than 30 DNA mutations transcribed in RNA. Thus, some less identifiable RNA editing events and novel mutations rescued by RNA can easily twist GBM’s case-level precision but are hard to affect GBM’s general precision. In addition, LUSC, BLCA, and GBM reached a median recall of 0.905, 0.880, and 0.857, concordant with their general recall. Also, RNA somatic mutation counts were highly correlated with DNA (Pearson correlation coefficient: LUSC: 0.905, BLCA: 0.937, and GBM: 0.607, Supplementary Figure S2) after excluding outlier cases with extreme mutation counts, suggesting the high accuracy of our framework.

We investigated the contributions of 37 features using an importance plot based on Gini impurity (Figure 4G) which showed that STRANDQ was the most important feature for discriminating RNA somatic mutations, followed by AF_tumor, TLOD, ROQ, and ECNT with nontrivial feature importance scores. In addition, features containing other sequencing qualities and population allele frequencies also played a role in prediction because they represented mutations’ reliability and germline evidence. We found that the prevalent RNA editing allelic changes “A>G” came at the bottom of the importance list, which indicated that our multi-filtering strategy adequately removed these editing sites and reduced their influence. Furthermore, we, in detail, illustrated the effects of 37 features on the prediction model by SHAP (Muyas et al., 2020) and ascertained whether their variations lowered or lifted the predicted probability (Supplementary Figure S3). After feature selection, we excluded “A>C,” “A>T,” and “MMQ_alt” features. Among all allelic change features, “A>G,” “C>A,” “C>G,” and “G>A” were retained. Out of which, “A>G” and “G>A” represented A-to-I (Wang et al., 2021) and C-to-U (Lerner et al., 2019) RNA editing events, and their existence had negative impacts on the model output. On the contrary, “C>A” and “C>G” represented RNA-editing exclusive allelic changes that exhibited positive impacts. Interestingly, we also found that high tumor allele depth for reference base and alternative base had opposite impacts, which indicated that RNA somatic mutations with high reference allele depth or low alternative allele depth in the tumor sample tended to be artifacts.

### Applications

#### Evaluation With Known DNA Evidence

We compared RNA-level somatic mutations with DNA-level to investigate the biological mechanisms for their intersection and uniqueness. As a result, we made a tabular overview (Table 3) and Venn diagrams (Supplementary Figure S4) to illustrate detailed distribution for the combination of RNA and DNA-level somatic mutations. Here, our framework successfully identified authentic mutations from the RNA-only part (which got ignored/not covered in WES data) to increase information gain and improve diagnostic yield. For all three parts, the RNA-only part had the largest mutation counts. The vast majority were labeled as negative (97.7–99.2%), indicating that our framework had successfully identified most artifacts in RNA because all these negative calls shall be filtered in the final output. Interestingly, when comparing mutation counts of the DNA-only part with the RNA–DNA overlap part, we found that less than 1/3 DNA somatic mutations got expressed in RNA. Such phenomenon was concordant with another study, mainly due to insufficient sequence coverage in low-expression or un-expression genes (Yizhak et al., 2019). Further analysis was listed in the following section for elaborate explanations.

**TABLE 3:** Overview of RNA somatic mutations combined with DNA.

Cancer type	RNA initial	RNA DNA overlap		RNA only		DNA only	P–R
		Positive	Negative	Positive	Negative		
LUSC	721,234	49,527	5,873	6,963	658,871	105,644	0.877–0.894
BLCA	323,323	43,945	6,557	6,206	266,615	71,614	0.876–0.870
GBM	126,449	9,153	1,947	970	114,379	16,104	0.904–0.825

Notes: Cancer type—LUSC: lung squamous cell carcinoma, BLCA: bladder urothelial carcinoma, GBM: glioblastoma multiforme.

DNA only—Counts of mutations only observed in the GDC DNA mutation set (not in RNA).

RNA–DNA overlap—Counts of mutations observed in both GDC DNA mutation set and RNA mutation set.

RNA only—Counts of mutations only observed in the RNA somatic mutation set (not in DNA).

RNA total—Counts of mutations observed in the total RNA somatic mutation set.

P–R—Precision–recall metric for RNA somatic mutations with GDC mutations as a golden standard dataset.

#### Variably RNA-Expressed Mutations Harbored a Special Enrichment Pattern

**FIGURE 5:**
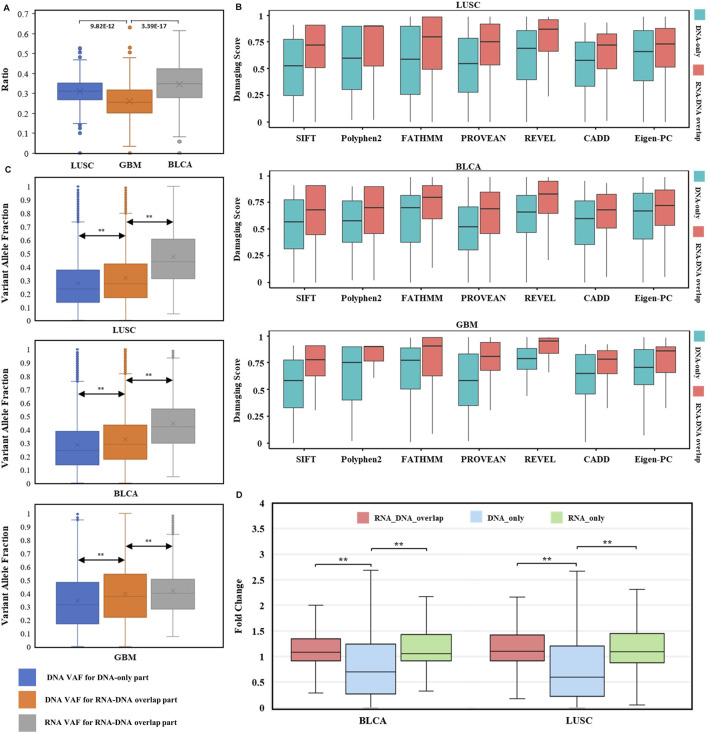
Evaluation of RNA somatic mutations and integrative analysis with DNA evidence. **(A)** Distribution of RNA expression ratios for known DNA somatic mutations across three types of cancer. Box plots’ heights ranged from 0.000 to 0.632. The comparisons utilized two-sided independent *t*-test with *p*-value < 1e-5. **(B)** Distributions of seven pathogenicity prediction scores for missense mutations within cancer driver genes across three cancer types (LUSC, BLCA, and GBM). DNA-only and RNA–DNA overlap parts in each cancer type were used for comparison (all comparisons passed two-sided independent *t*-test with *p*-value < 1e-5). **(C)** Variant allele fraction (VAF) distributions of DNA-only and RNA–DNA overlap parts within three cancer types. Left box: VAF distribution for DNA somatic mutations in DNA-only part. Middle box: VAF distribution for DNA somatic mutations in the RNA–DNA-overlap part. Right box: VAF distribution for RNA somatic mutations in the RNA–DNA-overlap part. The comparisons utilized two-sided independent *t*-test with *p*-value < 1e-5. **(D)** TPM fold change (FC) distributions for BLCA and LUSC. The comparisons utilized the Wilcoxon rank-sum test with *p*-value < 1e-5.

In detail, we explored the RNA expression ratios (number of expressed DNA somatic mutations/number of all DNA somatic mutations) for each case of three cancer types (Figure 5A), median expression ratios for LUSC, BLCA, and GBM were 0.312, 0.349, and 0.256, respectively. Highly variable expression ratios (0.000–0.632) in three types of cancer suggested that different DNA somatic mutations had various expression statuses in RNA. Notably, although the brain has a high number of expressed genes than other human tissues (Naumova et al., 2013), expression ratios of GBM were still significantly lower than those of LUSC or BLCA. These results indicated that DNA somatic mutations might be variably expressed or not expressed at all, and RNA somatic mutations were important to evaluate possible expression status.

To investigate whether the RNA-expressed somatic mutations tended to have larger functional impacts than those that only existed in DNA, probably resulting from the positive selection of cancer subclonal, we compared the impact scores of mutations within cancer-specific driver genes (Martinez-Jimenez et al., 2020) between RNA–DNA overlap and DNA-only parts. Interestingly, the cancer driver genes’ mutations were enriched in the RNA–DNA overlap part (LUSC: OR = 2.01, *p* = 1.14 e-68, BLCA: OR = 2.57, *p* = 9.89 e-119, GBM: OR = 2.70, *p* = 1.73 e-16. Supplementary Table S2), even though the DNA-only part had excessive mutation counts than the RNA–DNA overlap part (DNA-only/RNA–DNA overlap: ∼2/1). Moreover, we compared the predicted pathogenicity scores for missense mutations located within cancer driver genes between RNA–DNA overlap and DNA-only parts, and found that all RNA–DNA overlap parts had significantly higher pathogenicity scores across three cancer types and seven prediction tools (*p*-value < 1 e-5, Figure 5B). The significantly higher prediction scores implied that predicted damaging mutations tended to be selectively expressed in driving tumorigenesis, and our RNA-level somatic mutation identification framework effectively enriched the functional mutations.

Furthermore, we want to explore whether actionable mutations tend to get expressed in RNA and exhibit clinical effects. Thus, we assessed the therapeutic power for mutations in cancer driver genes between RNA–DNA overlap and DNA-only variants using the OncoKB database (Chakravarty et al., 2017) (https://www.oncokb.org/, Supplementary Table S3). Therapeutic sites within the RNA–DNA overlap part were far more than DNA-only across three cancer types (LUSC: OR = 13.34, *p* = 8.35e-19, BLCA: OR = 3.27, *p* = 4.26e-16, GBM: OR = 4.26, *p* = 3.66e-4, Table 4), indicating that the RNA-level somatic mutations calling can enrich clinical therapeutic variants. Notably, we observed that some therapeutic mutations from the OncoKB database also occurred in the DNA-only part. For example, except for 52 RNA–DNA overlap somatic mutations in BLCA, PIK3CA also had 12 DNA-only somatic mutations with “Level_3B″ OncoKB annotation (Chakravarty et al., 2017). We found that even if the 12 TCGA BLCA cases containing 12 DNA-only somatic mutations had sufficient expression level for the PIK3CA gene (TPM: 23.7–51.7, curated from UCSC Xena(Goldman et al., 2020) dataset), the 12 mutations’ alternative allele still got un-expressed (median alt allele-depth: 0) leading to unlikely benefit from certain targeted therapies. Therefore, although PIK3CA is a valuable therapeutic target for inhibitors of PI3K/AKT/mTOR pathways in advanced bladder cancer (Ross et al., 2016; Willis et al., 2020), the detailed expression status of the mutations should be carefully evaluated when the targeted therapy is considered. Such phenomenon was opposed to the assumption that mutations located within sufficiently expressed genes had undoubtful effects making them potential therapeutic targets, and RNA-level mutations were required to validate these targets’ transcription status.

**TABLE 4:** Overview of therapeutic mutation distribution in three types of cancer.

Therapeutic level	LUSC		BLCA		GBM	
	RNA–DNA overlap	DNA only	RNA–DNA overlap	DNA only	RNA–DNA overlap	DNA only
Level_1 FDA-approved drug	0	0	43	1	0	0
Level_2 standard care	0	0	0	0	1	0
Level_3 clinical evidence	58	3	140	40	13	1
Level_4 biological evidence	85	10	96	22	28	7
Counts sum	143	13	279	63	42	8
Total	1,240	1,333	1,565	1,014	175	116
OR (*p*_value)	13.34 (8.35 e-19)		3.27 (4.26 e-16)		4.26 (3.66 e-4)	

Notes: Level_1: FDA-recognized biomarker predictive of response to an FDA-approved drug.

Level_2: Standard care biomarker recommended by the NCCN predictive of response to an FDA approved drug.

Level_3–3A: Compelling clinical evidence supports the biomarker as being predictive of response to a drug; 3B: standard care or investigational biomarker predictive of response to an FDA-approved or investigational drug.

Level_4: Compelling biological evidence supports the biomarker as being predictive of response to a drug.

Counts sum: Sum of therapeutic mutation counts.

Total: Total counts for mutations located within cancer-specific driver genes.

Given that RNAs were enriched with mutations of higher functional impact and therapeutic value, we assessed the performance of RNA-level somatic mutations for discovering cancer driver genes by other statistical methods. Here, WITER (Jiang et al., 2019) was adopted to test the enrichment of somatic mutations due to positive selection in tumorigenesis (Jiang et al., 2019; Martinez-Jimenez et al., 2020). We compared the significant genes based on RNA-level somatic mutations to those based on the DNA-level somatic mutations in three cancer datasets. Among all significant genes (*FDR* < 0.1), the RNA somatic mutations led to a higher proportion of known cancer driver genes from the Intogen database (Martinez-Jimenez et al., 2020) in two of the three datasets than DNA (LUSC: 6/7 *vs*. 6/9 and GBM: 5/5 *vs*. 5/18, see details in Supplementary Tables S4, S5) with identical cancer-driver genes, another cancer type (BLCA: 12/18 *vs*. 15/19) also had a similar proportion. This result suggested that the RNA-level may lead to fewer false-positive estimations for driver genes than DNA-level.

In addition to known cancer driver genes, other significant genes based on the RNA-level somatic mutations, though un-registered in the Intogen database, were also functionally important to cancer development. CTNNB1, for example, had a significant *q*-value of 0.081 in BLCA. CTNNB1’s mutations have been found to cause aberrant WNT/CTNNB1 signaling and are associated with the susceptibility and prognosis of breast, endometrial, and gastric cancers (Kurnit et al., 2017; van Schie and van Amerongen, 2020; Wang et al., 2012). CHEK2 (*q* = 0.087 in LUSC, *q* = 0.052 in BLCA) played an important role in the repair of DNA damage, and its heterozygous mutations had been found to be causing genetic susceptibility to lung cancer (Wang et al., 2014) and bladder cancer (Złowocka et al., 2008). Although our detected CHEK2 somatic mutations were not inherited or passed on, their heterozygosity was similar and induced cancer risk. In a word, RNA can also prioritize potential cancer driver genes.

#### RNA Increased Mutation Detection Power.

VAF (variant allele fraction) was the fraction of sequencing reads harboring the mutation when performing NGS (Friedlaender et al., 2021), measuring the subclonal prevalence of specific mutations (Benard et al., 2021). We compared the DNA VAF distribution for DNA-only and RNA–DNA overlap parts within three cancer types to examine the subclonal selection advantage for expressed mutations. Higher DNA VAF was observed in expressed DNA somatic mutations (Figure 5C left comparison, *p* < 1 e-5), indicating the trend of cancer evolution for subclonal harboring RNA somatic mutations. Interestingly, RNA VAF was significantly higher than DNA VAF within expressed mutations of RNA–DNA overlap part (Figure 5C right comparison, *p* < 1 e-5), suggesting an expression tendency for the mutant allele. The common cancer WES study has a mutation limit of detection (LoD) at 5% VAF, and reporting these subclonal mutations incurs the risk of sequencing error–induced false positives (Yan et al., 2021). For these low-VAF (<0.05) DNA somatic mutations, their RNA VAFs were much higher, with median values of 0.374 in LUSC, 0.342 in BLCA, and 0.241 in GBM. Therefore, RNA somatic mutations exhibited subclonal selection superiority and increased the power for low-VAF mutation detection.

Here, we, in detail, demonstrated the recovery of DNA-omitted mutations for our framework. For the RNA-only part, we found that our framework helped rescue ∼10% of mutations (Table 3) which were missed based on DNA sequencing data. Most of the rescued mutations had low alternative allele depth (median: 0–1) or alternative allele fraction (median: 0–0.03) in WES data but opposite situations (median alt allele depth: 8–10, median alt allele fraction: 0.31–0.67) in RNA-seq data. There were also 102, 120, and 8 mutations located within cancer driver genes out of 6,997, 6,233, and 969 positive mutations from LUSC, BLCA, and GBM, respectively (Supplementary Table S6). Furthermore, we discovered biologically important cancer variants within these overlooked “driver” mutations using the DoCM database (Ainscough et al., 2016) (http://docm.info). We found that 17 out of 102, 14 out of 120, and 2 out of 8 DNA-overlooked “driver” mutations in LUSC, BLCA, and GBM had literature support from one or more publications (Supplementary Table S7). For example, TCGA-FD-A5BS had TP53 p.R282W mutation rescued by RNA with its reference-alternative allele depth in DNA: 19-1, RNA: 17-14. The R282W mutant had been found to cause the gain of novel oncogenic functions (GOF) in p53 proteins and associate with poorer cancer outcomes with a more prominent GOF effect (Zhang et al., 2016).

Low tumor purity can bias somatic mutation detection with the positive correlation between mutation numbers and tumor purities (Cheng et al., 2020). For example, TCGA-90-6837 in LUSC with its CPE (Aran et al., 2015) (consensus measurement of purity estimations) lower than average (0.56 vs. 0.68) had no official DNA mutation (WES failed to detect), we investigated its RNA somatic mutations identified by our framework to confirm its mutational status. We found that out of its 192 RNA somatic mutations, six mutations fell within cancer driver genes, and their existence had been ignored by WES (Table 5). Among these mutations, KMT2D is a lung tumor suppressor gene (Alam et al., 2020), and its mutation was one of the most significant prognostic factors in LUSC(Ardeshir-Larijani et al., 2018). We found that KMT2D p.E869∗ mutation could cause its truncation leading to tumor progression. In addition, TP53 p.A276G mutation had been found to locate within the DNA binding domain of the TP53 protein and presumably have deleterious impacts on protein functions (Chang et al., 2021) with pathogenic ClinVar database (Landrum et al., 2020) interpretation (Accession: VCV000185319.3). These findings confirmed that RNA-seq data could provide valuable supplementary information useful for clinical decisions and improve diagnostic yield in extreme cases when DNA failed to detect actionable mutations.

**TABLE 5:** RNA somatic mutation within cancer-driver genes in TCGA-90-6837.

Mutation	Gene	RNA		DNA		Protein change
		RefDepth	AltDepth	RefDepth	AltDepth	
chr4:186633790 T>C	*FAT1*	4	27	95	0	K1406R
chr8:116866708 G>A	*RAD21*	45	36	36	0	L8F
chr12:49051078 C>A	*KMT2D*	12	7	73	1	E869*
chr17:7673793 G>C	*TP53*	36	64	23	0	A276G
chr19:33026624 G>A	*RHPN2*	18	6	87	0	T65I
chr22:41178035 G>A	*EP300*	78	51	54	0	Q2108Q

#### Transcriptome-Wide Allele-Specific Expression Analysis

We calculated the TPM fold change (FC) to measure gene differential expression status. After excluding infinite FC values, we found that the median gene FC for RNA-expressed mutations was significantly higher than unexpressed mutations (Figure 5D). Thus, genes harboring RNA-expressed somatic mutations tended to have higher expression level in tumor samples than in paired normal samples.

We detected somatic SNV-level ASEs, and found that 24.8% of 3876 and 23.2% of 1700 somatic mutations exhibited ASE events in LUSC and BLCA RNA–DNA overlap parts. As expected, most (∼90%) ASE somatic mutations had over-expressed mutant alleles. The results showed that certain expressed somatic mutations had higher expression superiority in the mutant allele than the wild allele, which further enhanced the mutation detection power in RNA. Furthermore, we curated gene lists for 10 signaling pathways in cancer (Sanchez-Vega et al., 2018) and explored the functional alteration on signaling pathways for ASE somatic mutations. Ideally, if the ASE somatic mutation is functional, the direction of ASE event for the mutant allele should be the same as the direction of gene expression alteration for tumor vs. paired-normal samples (Liu et al., 2018). Thus, we mapped ASE somatic mutations to genes involving cancer signaling pathways with identical expression change direction. Finally, we identified several pathways (cell cycle, HIPPO, RTK RAS, TGF-Beta, and WNT) containing heavily altered genes with ASE events (Supplementary Table S8). Interestingly, seemly “benign” synonymous mutations also contained ASE events and altered gene expression level. For example, NF1 is a tumor suppressor that negatively regulates RAS signaling (Redig et al., 2016). NF1 p.L43L mutation in TCGA-39-5040 had an over-expressing mutant allele (DNA VAF: 0.32, RNA VAF: 0.63) and showed an upregulated gene expression (tumor/paired-normal fold change: 2.53), which activated NF1 function to under-regulate the RAS signaling pathway and suppressed carcinogenesis.

## Discussion

Although common somatic mutation detection practices come with WES, important and actionable mutations are often conserved in RNA-seq. Therefore, we developed RNA-SSNV, an integrative framework to identify RNA somatic single nucleotide variants called within tumor RNA-seq and paired-normal WES data. To maximize performance, we combined multi-filtering strategies and a machine-learning model. For the multi-filtering strategy, we found that it removed massive artifacts (∼70%) while omitting few true positive calls (∼0.1%). Before constructing the classification model, we also evaluated the performance of the GATK-recommended filtering tool (FilterMutectCalls) for the LUAD training dataset and LUSC, BLCA, and GBM validating datasets using precision–recall metrics. The result showed that FilterMutectCalls achieved a satisfying recall but a low precision rate (LUAD P–R: 0.380–0.865, LUSC P–R: 0.399–0.871, BLCA P–R: 0.442–0.886, and GBM P–R: 0.540–0.881), which may lead to large false-positive calls. Because FilterMutectCalls was originally designed based on DNA somatic mutation filtering strategy, which may not be fully compatible with RNA, we adopted a machine learning model with comprehensive features to conduct classification. For model training, we adopted various techniques to ensure its reliability. To construct a high-quality training dataset, we used GDC DNA mutations as the golden standard and self-called DNA mutations as important supplementary information to separate pure true positive and true negative sets from multi-filtered RNA mutations. In a comparison of using two data sources (RNA mutations and golden-standard DNA mutations) to construct the training dataset, the introduction of self-called DNA mutations significantly improved our machine model’s performance (increased precision–recall from 0.843-0.875 to current 0.883–0.885 by 4%). We also conducted feature selection and fine-tuning to improve the model’s performance. Eventually, our trained model achieved superior performance of 88.0% precision and 88.4% recall rate in the testing dataset compared with other state-of-art RNA somatic mutation detection tools such as RNA-Mutect (Yizhak et al., 2019) (precision: 0.87, recall: 0.72) and RF-RNAmut (Muyas et al., 2020) (precision: 0.85, recall: 0.71).

When utilized in independent validation datasets (TCGA LUSC, BLCA, and GBM), RNA-SSNV achieved similar performance as in the testing dataset, which had 0.871–0.895, 0.876–0.871, and 0.902–0.830 precision–recall rate, respectively. Not only can our framework reliably detect RNA somatic mutations, but it also can conduct pairwise analysis with provided DNA mutations. Although our framework achieved satisfying performance within somatic RNA single-nucleotide variants’ identification, limited scenarios in which only RNA somatic mutations can be retrieved such as the GTEx project (Lonsdale et al., 2013) (contained RNA-seq data from ∼6700 samples across 29 normal tissues). Common RNA-seq practices involving research always included DNA-seq data which generated somatic DNA mutations simultaneously; thus, the investigation for the relationship between DNA-level and RNA-level somatic mutations was essential. Multiple studies have found that combining DNA-level and RNA-level somatic mutation can achieve maximum performance for mutational investigation (Krug et al., 2018; Newman et al., 2021; Wilkerson et al., 2014; Zhang et al., 2020). Thus, we split DNA and RNA somatic mutations into three parts: DNA–RNA overlap part, DNA-only part, and RNA-only part; and each part had positive and negative sub-parts representing our model’s classifications. The DNA–RNA overlap part represented orthogonal validated DNA and RNA mutations; its positive sub-part contained reliable cancer somatic mutations with clinical usage, but its negative sub-part contained false-negative calls misclassified by our model. When using SHAP to analyze these false-negative calls (Supplementary Figure S5), we found that G>A mutant status had significant impacts, which implicated that APOBEC-mediated C-to-U RNA editing events (Lerner et al., 2019) contributed to misclassification and current RNA editing resources were insufficient to filter C>U editing sites. DNA-only part represented DNA mutations omitted in RNA somatic mutation calling, and we found that some DNA mutations’ reference allele got selectively expressed while their alternative allele got silenced. To explore how many DNA-only somatic mutations got selectively expressed, we calculated the selective expression ratios (number of mutations with reference allelic depth>10/number of DNA somatic mutations not identified in RNA) for DNA-only parts across three cancer types (Supplementary Figure S6). The median mutation selective expression ratios for LUSC, BLCA, and GBM were 0.134, 0.120, and 0.154, respectively, confirming that DNA somatic mutations within GBM had higher selective expression tendency than LUSC (*p* = 0.003) and BLCA (*p* = 5.63 e-6), possibly due to innate upregulation of DNA repair mechanisms (Ferri et al., 2020). We retrieved their information in RNA using Mutect2’s force-calling mode and utilized our model to classify them. Most of them were predicted negative as expected, but a small portion (1.9%) was predicted as positive, suggesting that our selected caller (Mutect2) might have a little neglection. We also observed that mutations’ reference allele–specific expression within driver genes leads to doubtful translation effects. In addition, most mutations located within collagen-related genes (COL11A1, COL6A3, COL5A2, etc.) were found silenced while these genes got sufficiently expressed in RNA (Supplementary Table S9). Interestingly, the proteome database (Human Cancer Proteome Variation Database) also contains nearly no evidence for mutant collagen proteins across three cancer types which were abnormal because massive DNA somatic mutations had been found in these genes. The RNA-only part represented RNA mutations without DNA evidence support. Its negative sub-part was artifacts, but its positive sub-part included RNA-rescued mutations missing in DNA that contained mutations within cancer driver genes (1.4%) to provide more therapeutic targets and help with clinical decisions. A major shortcoming of WES is uneven coverage of sequence reads over the exome targets contributing to many low-coverage regions (Wang et al., 2017; Xiao et al., 2021), and substantial inter-individual variation in coverage of medically implicated genes caused false-negative mutation calls due to low coverage (Barbitoff et al., 2020; Kong et al., 2018). Although using replicate exome-sequencing can improve WES coverage by 4.3–12.7% (Cherukuri et al., 2015), improve variant calling accuracy (Zhang et al., 2014), and enhance clinical interpretation, information redundancy and excess costs limited its usage. Compared with replicate exome-sequencing, RNA-seq has improved somatic single nucleotide variants, and clinically actionable mutations are often conserved in RNA.

We also examined the potential of improving our model’s performance by introducing additional training data from different cancer types. After adding GBM cancer–type data into the training dataset, we only observed a slight improvement within the testing dataset (recall rate increased 1.3%) and the AUC for P–R curves for TCGA LUSC, BLCA–independent validation datasets remained stable at 0.94 (Supplementary Figure S7). The unchanged performance suggested that our model trained with LUAD datasets probably has already contained key features of RNA somatic mutation in cancer cells and is applicable for other cancers. Although the general performance for our model was identical across three validation datasets, performances under default threshold (0.5) slightly differed and a dynamic shift of threshold according to different aims (prefer higher precision or recall) was required. In addition, due to insufficient C-to-U RNA editing database resources, the current model sacrificed high recall to ensure removing editing events for the G>A mutation type. The high distribution of G>A mutations (52.3%) in false-negative sets of TCGA LUSC–independent validation dataset reflected this imperfection. Therefore, we recommended that users manually review predicted-negative G>A mutations within known driver genes to improve diagnosis. To facilitate user to inspect predictions, we provided codes to visualize the contribution of important features using SHAP library and a canonical table to exhibit all useful information for user-specified records. A major limitation of our framework was that it was designed to identify RNA somatic mutations only from tumor RNA-seq and paired-normal WES data. Future works will include extending RNA somatic mutation identification scope into other sequencing data types (single-cell RNA-seq or whole-genome DNA-seq).

For cancer research involving both WES and RNA-seq data, the conventional analysis strategy uses WES data to call somatic mutations and then validates whether somatic mutations exist in RNA-seq data. However, the conventional strategy may still omit some somatic mutations in RNA-seq data. Our study significantly improved the capability to call RNA somatic mutations and further revealed the association between somatic mutations derived from RNA and DNA, providing valuable supplementary information for conventional cancer somatic mutation analysis.

## Data Availability

The original contributions presented in the study are included in the article/Supplementary Material; further inquiries can be directed to the corresponding author.
