# Supplementary material for: RNA-SSNV: A Reliable Somatic Single Nucleotide Variant Identification Framework for Bulk RNA-Seq Data
Source: Front Genet. 2022 Jun 30;13:865313. doi: 10.3389/fgene.2022.865313 (PMC9279659; doi:10.3389/fgene.2022.865313)
Supplement: Supplementary file 3 [file Presentation1.PPTX]

## Slide 1
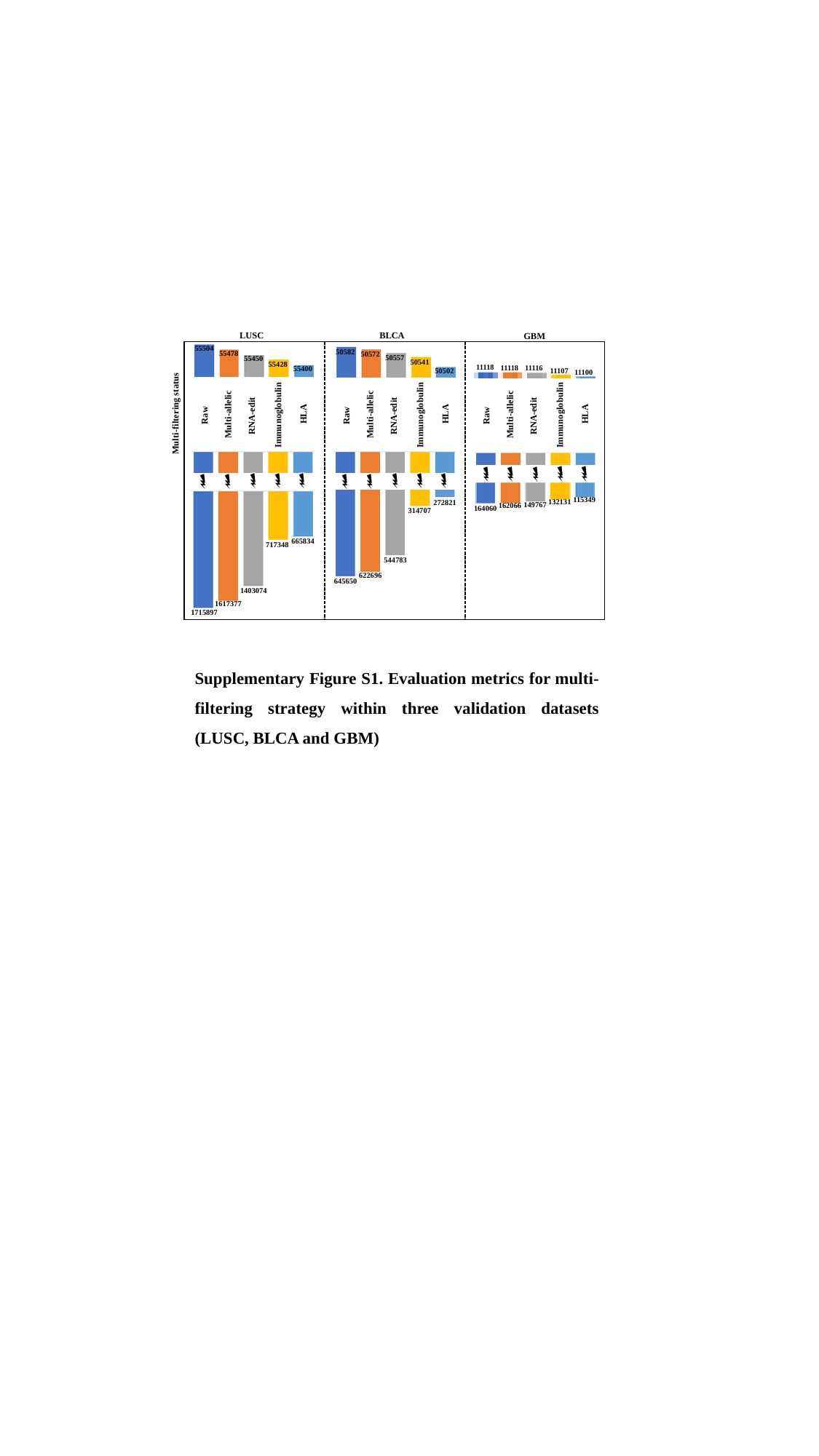

LUSC
55504
55478
55450
55428
55400
HLA
Raw
RNA-edit
Immunoglobulin
Multi-allelic
665834
717348
1403074
1617377
1715897
BLCA
50582
50572
50557
50541
50502
HLA
Multi-filtering status
Raw
RNA-edit
Immunoglobulin
Multi-allelic
272821
314707
544783
622696
645650
GBM
11118
11118
11116
11107
11100
HLA
Raw
RNA-edit
Immunoglobulin
Multi-allelic
115349
132131
149767
162066
164060
Supplementary Figure S1. Evaluation metrics for multi-filtering strategy within three validation datasets (LUSC, BLCA and GBM)

## Slide 2
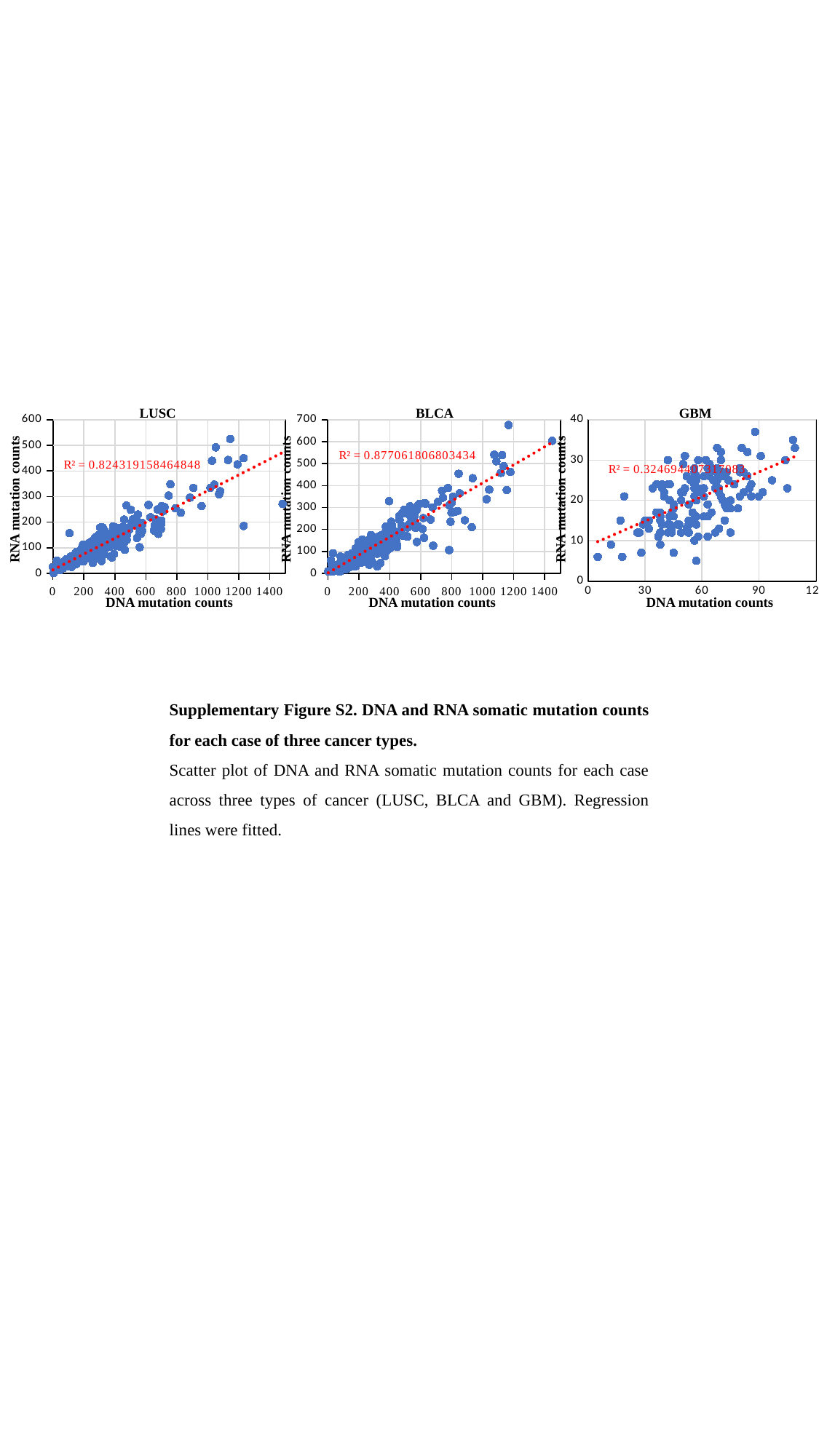

LUSC
BLCA
GBM
### Chart
| Category | Y 值 |
|---|---|
### Chart
| Category | Y 值 |
|---|---|
### Chart
| Category | Y 值 |
|---|---|RNA mutation counts
RNA mutation counts
RNA mutation counts
DNA mutation counts
DNA mutation counts
DNA mutation counts
Supplementary Figure S2. DNA and RNA somatic mutation counts for each case of three cancer types.
Scatter plot of DNA and RNA somatic mutation counts for each case across three types of cancer (LUSC, BLCA and GBM). Regression lines were fitted.

## Slide 3
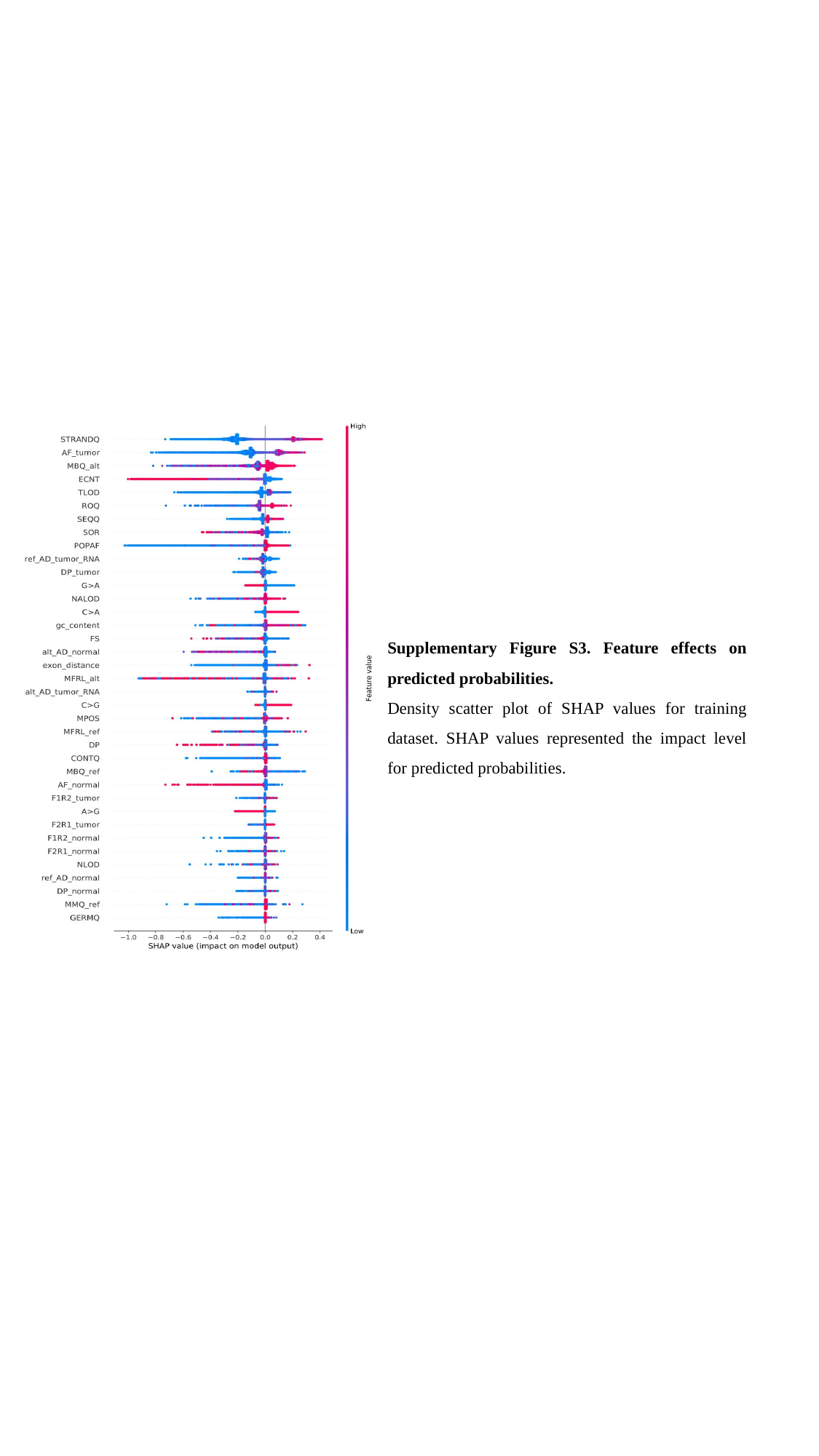

Supplementary Figure S3. Feature effects on predicted probabilities.
Density scatter plot of SHAP values for training dataset. SHAP values represented the impact level for predicted probabilities.

## Slide 4
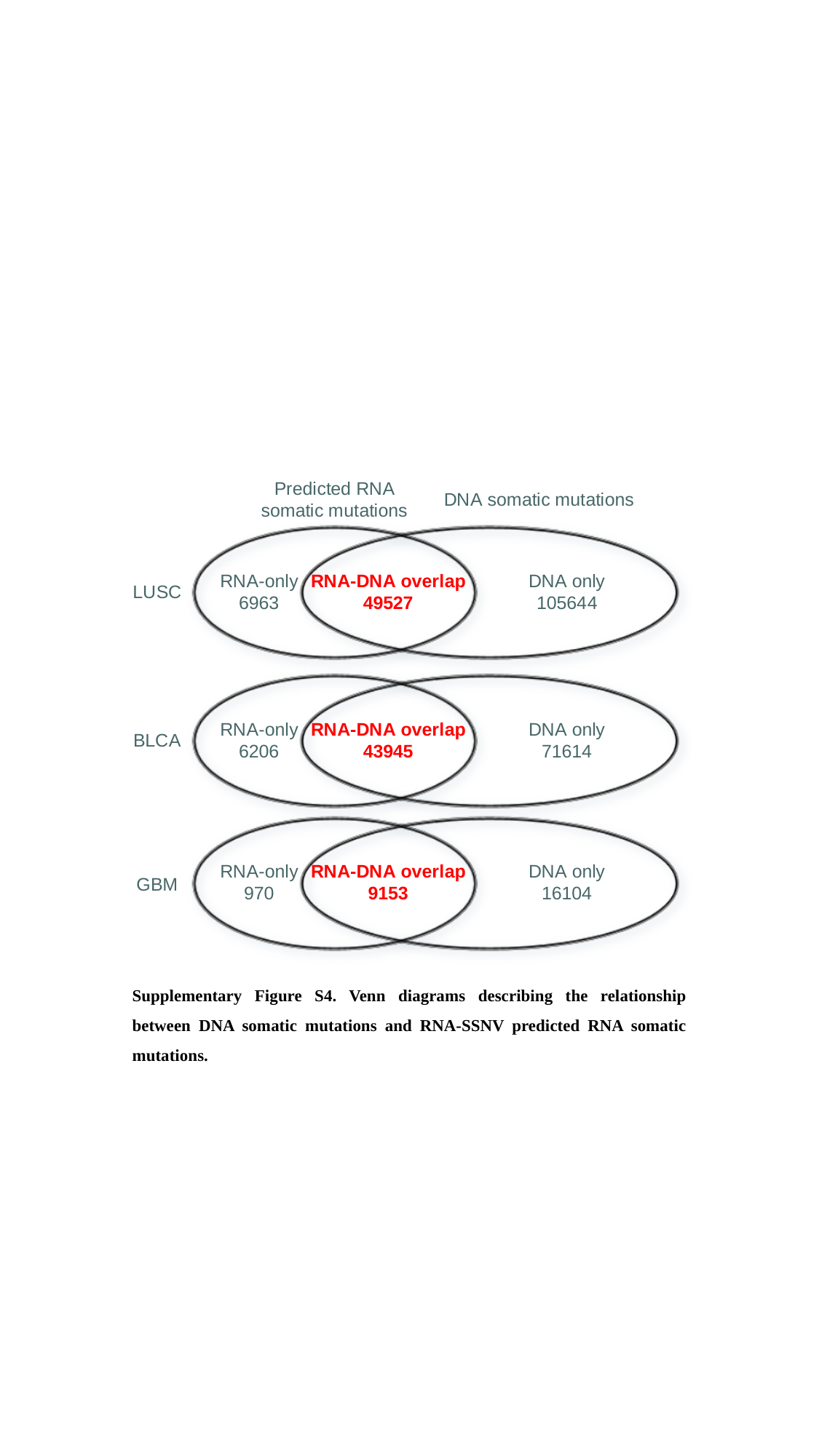

Supplementary Figure S4. Venn diagrams describing the relationship between DNA somatic mutations and RNA-SSNV predicted RNA somatic mutations.

## Slide 5
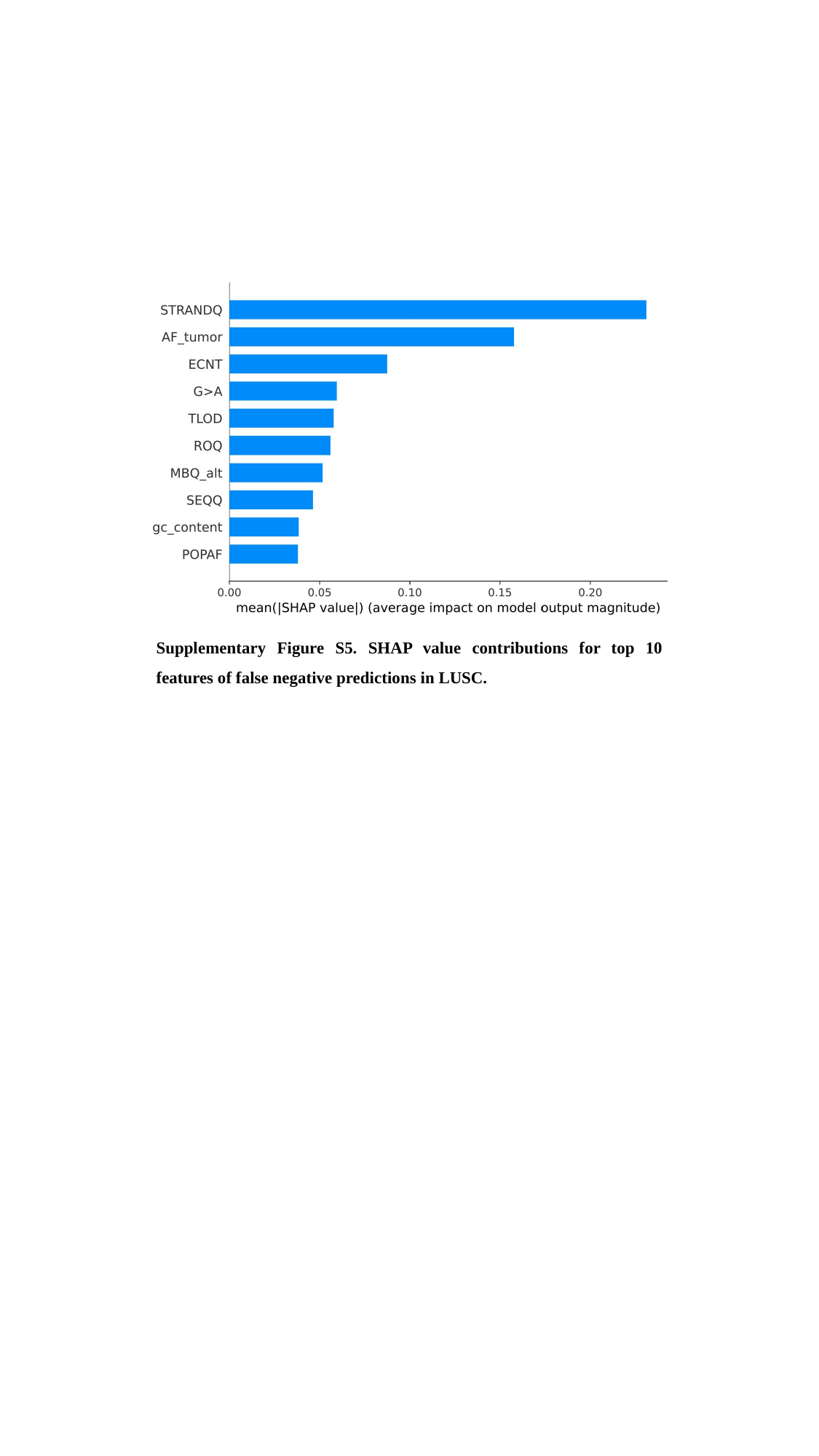

Supplementary Figure S5. SHAP value contributions for top 10 features of false negative predictions in LUSC.

## Slide 6
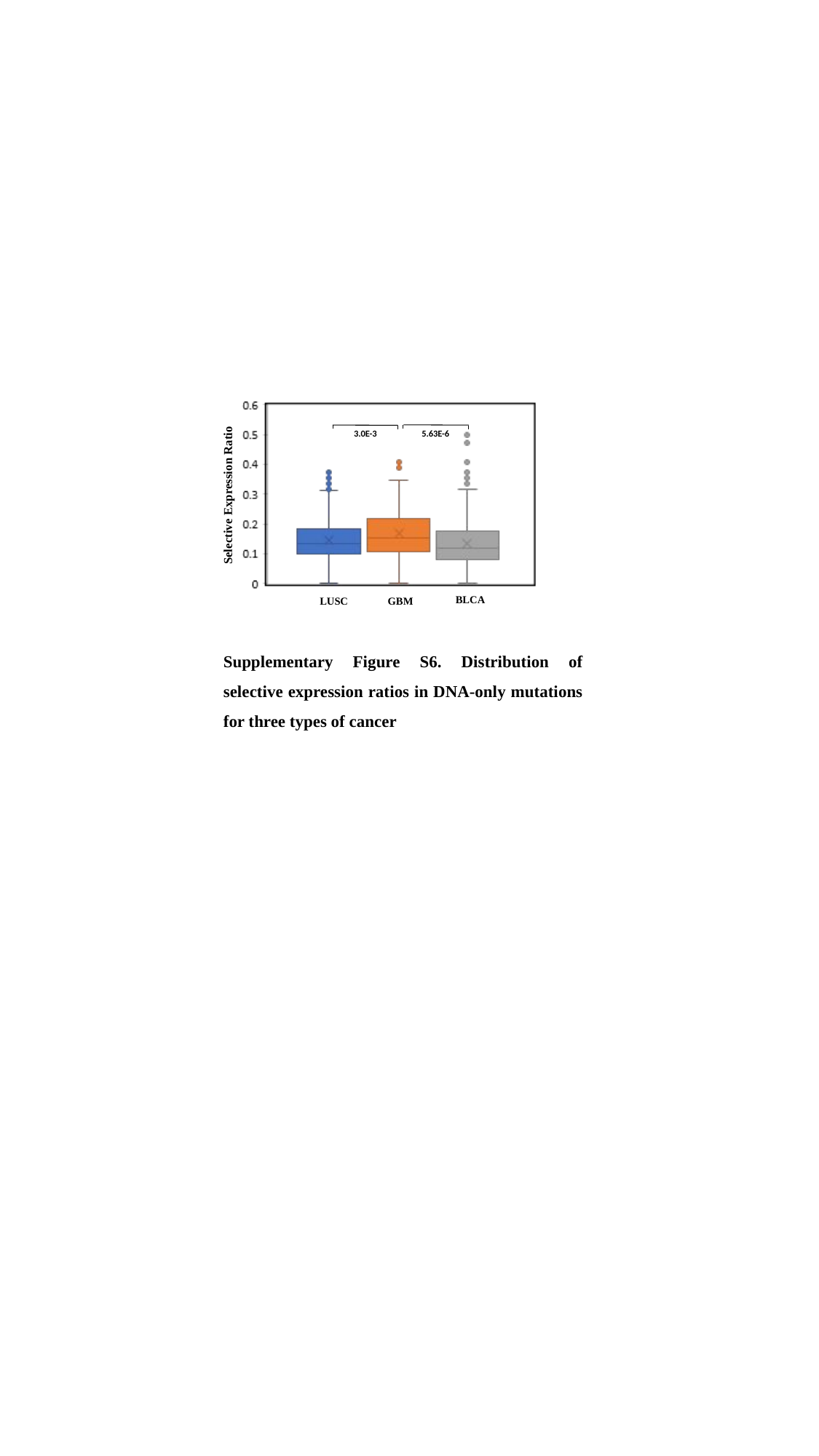

5.63E-6
3.0E-3
Selective Expression Ratio
BLCA
LUSC
GBM
Supplementary Figure S6. Distribution of selective expression ratios in DNA-only mutations for three types of cancer

## Slide 7
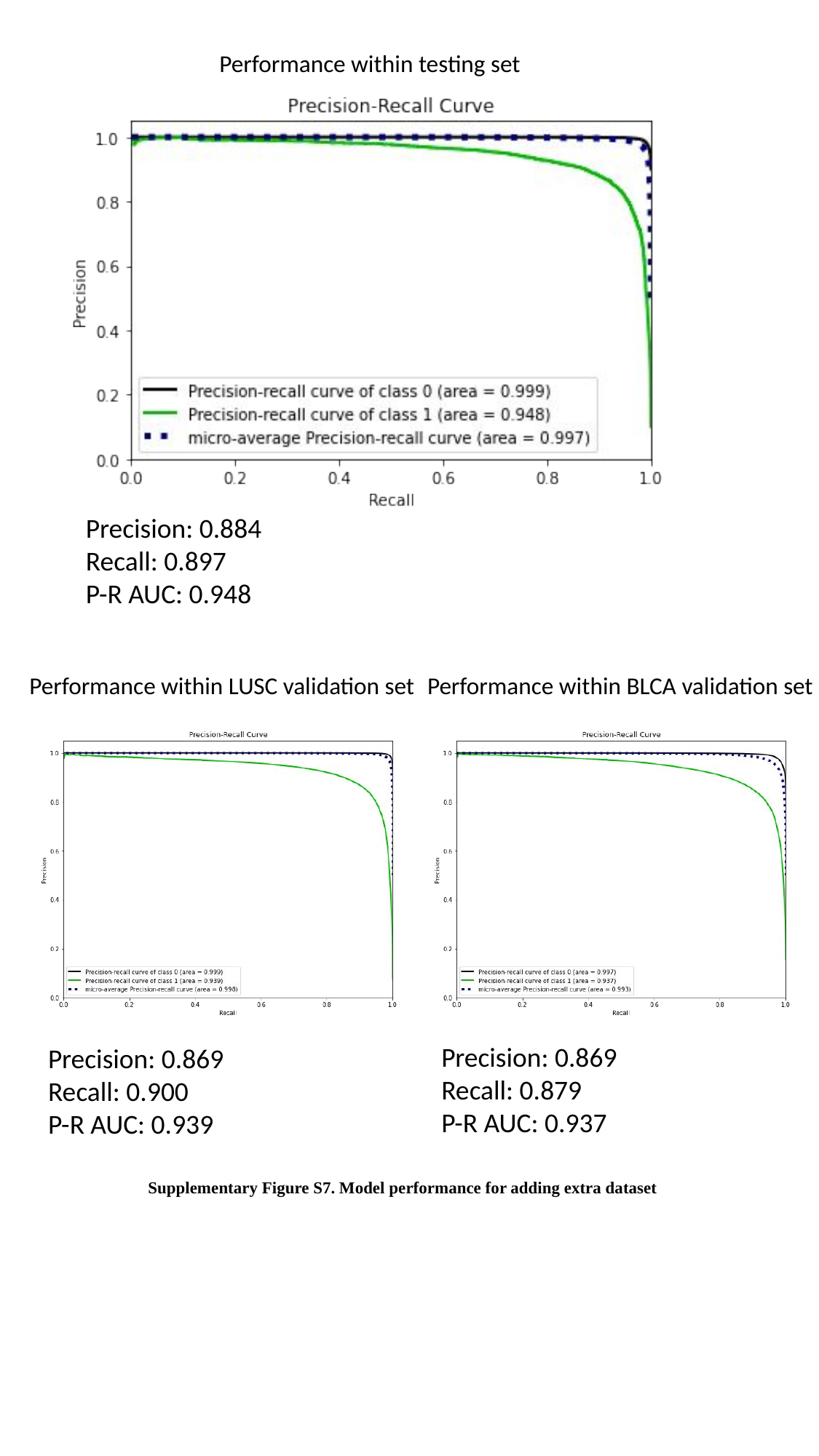

Performance within testing set
Precision: 0.884
Recall: 0.897
P-R AUC: 0.948
Performance within LUSC validation set
Performance within BLCA validation set
Precision: 0.869
Recall: 0.879
P-R AUC: 0.937
Precision: 0.869
Recall: 0.900
P-R AUC: 0.939
Supplementary Figure S7. Model performance for adding extra dataset
